# FRAILTY AND INTRINSIC CAPACITY IN OLDER ADULTS IN CHINA: FROM RESEARCH TO PRACTICE

**DOI:** 10.1093/geroni/igae098.1477

**Published:** 2024-12-31

**Authors:** Lina Ma

**Affiliations:** Xuanwu Hospital, Capital Medical University, Beijing, Beijing, China (People’s Republic)

## Abstract

Older adults with frailty have a reduced ability to cope with stresses, such as that associated with acute diseases, and these adults also have an increased risk of falls, disability, and death. The prevention and treatment of frailty in this group of people are very important. In China, as the general population has aged, the demand for medical care services for older adults has increased. China has given high priority to healthy aging and established a national strategy “Healthy China 2030” blueprint and is progressing rapidly in geriatric medicine. Although frailty research in China started late, it has developed very rapidly, especially on assessment tools, epidemiology, and management. Furthermore, several guidelines have been established to help physicians make clinical decisions while treating frail older persons. In recent years, clinicians and researchers have shown increasing interest in intrinsic capacity, a new positive approach to health aging. However, much effort should be made to integrate frailty into clinical practice. This talk focuses on the research advances on frailty from biological mechanisms to therapeutic opportunities as well as individual-centered integrated care for optimizing intrinsic capacity to prevent frailty and disability in older adults in clinical practice in China.

